# Purpose in Life and Character Strengths as Predictors of Health Sciences Students’ Psychopathology During the COVID-19 Pandemic

**DOI:** 10.3389/fpsyt.2022.932249

**Published:** 2022-07-05

**Authors:** Iván Echeverria, Marc Peraire, Danaide Penadés, Valentina Quintero, Ana Benito, Isabel Almodóvar, Gonzalo Haro

**Affiliations:** ^1^TXP Research Group, Universidad Cardenal Herrera-CEU, CEU Universities, Castelló de la Plana, Spain; ^2^Department of Mental Health, Consorci Hospitalari Provincial de Castelló, Castelló de la Plana, Spain; ^3^Faculty of Health Sciences, Universidad Cardenal Herrera-CEU, CEU Universities, Castelló de la Plana, Spain; ^4^Torrente Mental Health Unit, Hospital General de Valencia, Valencia, Spain

**Keywords:** COVID-19, character strengths, medical students, mental health, moral courage, nursing students, psychopathology, purpose in life

## Abstract

**Background:**

Health sciences students experience high levels of psychopathology conditioned by psychosocial, financial, and academic factors. However, COVID-19 pandemic might even have worsened their mental health. Thus, this article aims to evaluate how the exposure to COVID-19 pandemic has affected these students’ mental health and to determine the effect of purpose in life and character strengths on this psychopathology.

**Methods:**

A cross-sectional study of unpaired samples was carried out in Spain during the first and third waves of the pandemic in 70 medical and 52 nursing students.

**Results:**

The risk factor that most determined the appearance of anxiety was the exposure of family and friends to COVID-19 (OR = 4.01; *p* < 0.001), while the most protective factors were honesty (OR = –1.14; *p* = 0.025) and purpose in life (OR = –0.18; *p* < 0.001). Purpose in life also protected against the onset of depression and total psychopathology. In addition, we observed studying medicine was a protective factor against total psychopathology while being a nursing student was associated with high levels of acute stress.

**Conclusion:**

Exposure of the students’ family and friends to SARS-CoV-2 favored the appearance of symptoms of anxiety. Honesty had a preventing role in the onset of anxiety and a high purpose in life was protective against the appearance of anxiety, depression, and total psychopathology.

## Introduction

Studying health sciences acts as a risk factor for developing psychopathology, such as anxiety, depression, acute stress and alcohol or substance abuse (1). Starting university represents a series of vital changes for students, all of which must be managed with specific strategies to deal with these challenges appropriately. Problems arise when most students lack these strategies or when their strategies are inadequate (2).

During their academic training, medical, and nursing students experience high levels of distress which is conditioned by psychosocial, financial, and academic factors (3–5). This fact does not only means that students cannot get the most out of their training, but also affects other areas of their lives (6), including their mental health.

A recent analysis carried out by the State Council of Medical Students (CEEM in its Spanish acronym) concluded that up to 41% of medical students presented at least one sign of depression, and 24.7% experienced high levels of anxiety (5). In the same vein, some studies (3, 4) have shown that nursing students experience signs of depression in a similar proportion to those in the CEEM study, but that they also show much higher levels of anxiety, at around 50%, with women predominating in both groups. These figures indicate the growing appearance of psychopathology in future healthcare professionals, starting from the earliest moments of their training. In fact, it has been shown that many healthcare workers already suffer from anxiety and depression problems from the very beginning of their university education (2). In addition, alcohol and substance abuse is another issue related to the health sciences students, especially since the COVID-19 pandemic (7).

As seen, anxiety, depression, acute stress, and alcohol or substance abuse are considered very prevalent conditions in health sciences students. Because of this, an attempt has been made to identify possible predisposing factors so that effective prevention measures can be implemented (8). In this sense, it has been shown that stressful life situations, such as the current context of the COVID-19 pandemic, are very important factors for the emergence of these types of psychopathology (7, 9). The psychological impact of COVID-19 pandemic has recently been evaluated not only in health sciences students (10), but also in healthcare workers (11) and general population (12) worldwide.

Among university careers, medicine and nursing are two traditionally vocational choices, a fact that the virus has significantly highlighted (13). Students who choose one of these disciplines tend to do so because they were moved by a specific purpose in life (PIL), described as an individual’s perception of the objective and value of their life (11); character strengths, which are the positive parts of the core personality that impact how you think, feel and behave (14); and varying degrees of moral courage, defined as the ability to face danger or social disapproval when performing what one believes to be their duty (15). They develop their PIL, character and personal values through “holistic intelligence,” in other words by considering human beings as a whole, not only as something physical or psychological, but also considering moral integrity, service to others, and generosity (16). Although medical and nursing careers complement each other and must work together, there are several differences between the two (17–19); for instance, in terms of the education length, type of training received, and focus on care, care actions, professional–patient relationships, work self-management, and the need for specialization.

Research has been published on the psychopathological consequences of COVID-19 in students in general (20), but no scientific articles have specifically related the presence of symptoms with PIL, character strengths and moral courage among medical and nursing students. Thus, this study aimed to evaluate the possible predictor effect of these dimensions and to determine what other elements could help predict the appearance of psychopathology in health sciences students.

## Materials and Methods

This study had a cross-sectional, observational design and used two unpaired samples. A group of 70 medical students and another group of 52 nursing students from a Spanish university, all of whom were studying the last year of their respective degrees, were obtained through intentional sampling by sending the questionnaire to their e-mail and mobile phones. We used G*Power software to calculate the sample size required to evaluate the differences in the psychopathological variables between the two groups using *t*-test. For an effect of 0.5, with an alpha of 0.05, power of 0.80, and an N2/N1 allocation ratio of 1.3, a group size of minimum 59 for group 2 (medicine) and 45 for group 1 (nursing) would be required (total *n* = 104), so the sample recruited (70 medical students and 52 nursing students) is sufficient. The data were collected between 20 April and 27 May 2020 (2019–2020 academic year) when Spain was immersed in the peak of the first COVID-19 wave, and between 4 December 2020 and 19 January 2021 (2020–2021 academic year) during the third wave, with the latter having had the greatest generalized impact on society (21).

After signing their informed consent to participate, study participants completed a self-assessment using a series of instruments in Spanish.

First, they completed a questionnaire on sociodemographic variables.

Variables that evaluated psychopathology were considered as dependent variables. In the analyzes, total scores of the scales were used and dichotomous variables were created differentiating those that exceeded the cut-off point (CP) of each scale and those that did not. To assess anxiety, depression, and acute stress we used the Beck’s Anxiety Inventory (BAI) [CP = 8; reliability = 0.90; adequate factorial, discriminant and criterion validity (22)], Beck’s Depression Inventory (BDI) [CP = 14; reliability = 0.89; adequate factorial, convergent, discriminant, and criterion validity (23)], and an *ad hoc* questionnaire based on the DMS-5 criteria for assessing acute stress (reliability = 0.81), respectively. Drug abuse was assessed using the Drug Abuse Screening Test (DAST-10) [CP = 1; reliability = 0.89; proven predictive validity (24)] and alcohol abuse was examined using the Alcohol Use Disorders Identification Test (AUDIT) [CP for women = 6, and CP for men = 8 (25); reliability = 0.75; adequate criterion and predictive validity (26)].

Variables that evaluated the different types of exposure to COVID-19 (personal exposure and that of their family and friends) were considered as independent variables. A 2 item Likert like questionnaire used in a previous study (11) was administered. Total exposure was calculated by summing personal and family and friends exposure. PIL, character strengths and moral courage were also considered as independent variables. The former was analyzed using the PIL scale [CP = 113; adequate internal consistency and construct validity (27)], calculating a dichotomous variable that differentiated between those who presented a high sense of PIL and those who did not. Global Assessment of Character Strengths-24 (GACS-24) [reliability = 0.78 (28)] was used to assess the character strengths of the participants. Finally, moral courage was assessed with the Moral Courage Scale for Physicians (MCSP) [reliability = 0.90; proven factorial, convergent, discriminant and concurrent validity (29)] and the Professional Moral Courage Scale (PMCS) [construct validity achieved (30); reliability = 0.85; supported factorial and convergent validity (31)].

SPSS software (version 27) for Microsoft (IBM Corp., Armonk, NY.) was used for all the statistical analyses. After the exploratory (normality, independence, homoscedasticity, linearity, non-collinearity) and descriptive study, the variables were subsequently compared using Student’s t-distribution for quantitative variables and Pearson chi-squared tests for categorical variables. Linear regression models were created for the psychopathological variables, introducing exposure to SARS-CoV-2, PIL, character strengths and moral courage as independent variables. Finally, the data were modeled using the PROCESS plugin (v3.4) for SPSS (32).

The ethical principles set out in the Declaration of Helsinki and by the Council of Europe Convention were followed and the informed consent of all participants was obtained. Moreover, data confidentiality was guaranteed according to the General Data Protection Regulation (GDPR; 2018). This study was authorized by the Investigation Commission at the Provincial Hospital Consortium in Castellon (ref. A-15/04/20) and the Clinical Research Ethics Committee of the Cardenal Herrera-CEU University (ref. CEI20/068).

## Results

### Sociodemographic Characteristics

Regarding sociodemographic characteristics, most of the students were women, although this predominance was higher in the nursing student group (χ^2^ = 6.556; *p* = 0.010). Significantly more students in the medical student group were single (98.6%; *n* = 48) while more were married in the nursing student group (11.5%; *n* = 6; χ^2^ = 7.123; *p* = 0.028). Finally, there were significantly less smokers in the medical student group (11.4%; *n* = 8) than in the nursing student group (28.8%; *n* = 15; χ^2^ = 5.917; *p* = 0.015) (Table 1).

**TABLE 1:** Sociodemographic characteristics and differences between the study groups.

	Total *n* = 122	Nursing students *n* = 52	Medical students *n* = 70	
	% (*n*)/*M* (*SD*)	% (*n*)/*M* (*SD*)	% (*n*)/*M* (*SD*)	*t*/χ^2^(*p*) *post hoc*/CTR
Age	24.8 (4.842)	25.38 (6.350)	24.34 (3.292)	*t* = 1.080 (*p* = 0.284)
Sex				χ^2^ = 6.556 (*p* = 0.010)
Female	82.0 (100)	**92.3 (48)**	74.3 (52)	2.6/–2.6
Male	18.0 (22)	7.7 (4)	**25.7 (18)**	–2.6/2.6
Religiosity Yes	53.3 (65)	57.7 (30)	50.0 (35)	χ^2^ = 3.442 (*p* = 0.632)
Marital status				χ^2^ = 7.123 (*p* = 0.028)
Single	93.4 (114)	86.5 (45)	**98.6 (69)**	–2.7/2.7
Married	5.7 (7)	**11.5 (6)**	1.4 (1)	2.4/–2.4
Divorced	0.8 (1)	1.9 (1)	0.0 (0)	1.2/–1.2
Physical illness yes	13.1 (16)	7.7 (4)	17.1 (12)	χ^2^ = 2.339 (*p* = 0.126)
Smoker yes	18.9 (23)	**28.8 (15)**	11.4 (8)	χ^2^ = 5.917 (*p* = 0.015) 2.4/–2.4
Psychiatric history yes	14.8 (18)	11.5 (6)	17.1 (12)	χ^2^ = 0.745 (*p* = 0.388)
Psychological/Pharmacological treatment during quarantine yes	3.3 (4)	1.9 (1)	4.3 (3)	χ^2^ = 0.223 (*p* = 0.637)

*CTR, corrected typified residuals; those less than –1.96 or greater than 1.96 were considered significant. The groups from among the categorical variables in which the CTRs were significant are shown in bold. n, sample; M, average; SD, standard deviation; χ^2^, Pearson chi-squared test; t, Student’s t-test.*

### COVID-19 Exposure, Purpose in Life, Character Strengths, and Moral Courage

Table 2 shows that there were no differences in exposure to COVID-19 between these groups, either at a personal or a family and friends level. However, compared to the nursing students, medical students presented lower scores for the GACS-24 item about love (*M* = 5.87; *SD* = 1.382 vs. *M* = 6.38; *SD* = 0.844; t = 2.535; *p* = 0.013) and about teamwork (*M* = 5.89; *SD* = 1.123 vs. *M* = 6.37; *SD* = 0.768; t = 2.652; *p* = 0.009). In addition, we observed significantly higher PMCS score among medical students (*M* = 10.97; *SD* = 1.142) than in nursing students (*M* = 10.25; *SD* = 1.846; t = –2.487; *p* = 0.015).

**TABLE 2:** Exposure to SARS-CoV-2 and moderating variables.

	Total *n* = 122	Nursing students *n* = 52	Medical students *n* = 70	
	% (*n*) / *M* (*SD*)	% (*n*) / *M* (*SD*)	% (*n*) / *M* (*SD*)	*t*/χ^2^ (*p*) *post hoc*/CTR
Personal exposure	0.56 (0.980)	0.63 (1.067)	0.50 (.913)	*t* = 0.749 (*p* = 0.455)
Family/friends exposure	0.52 (0.633)	0.63 (.742)	0.43 (.527)	*t* = 1.709 (*p* = 0.091)
Personal and family/friends exposure	1.07 (1.200)	1.26 (1.285)	0.93 (1.120)	*t* = 1.559 (*p* = 0.122)
PIL	110.18 (14.401)	111.44 (14.549)	109.24 (14.323)	*t* = 0.833 (*p* = 0.406)
PIL yes	50 (61)	59.6 (31)	42.9 (30)	χ^2^ = 3.352 (*p* = 0.067)
GACS-24	136.84 (15.490)	137.67 (14.230)	136.21 (16.437)	*t* = 0.513 (*p* = 0.609)
Creativity	5.10 (1.256)	5.17 (1.184)	5.04 (1.313)	*t* = 0.565 (*p* = 0.573)
Curiosity	5.92 (1.103)	5.94 (1.018)	5.90 (1.169)	*t* = 0.209 (*p* = 0.835)
Critical thinking	5.65 (1.113)	5.58 (1.016)	5.70 (1.184)	*t* = –0.603 (*p* = 0.548)
Passion for learning	5.98 (1.195)	5.98 (1.229)	5.97 (1.179)	*t* = 0.043 (*p* = 0.966)
Wisdom	5.82 (1.021)	5.65 (0.988)	5.94 (1.034)	*t* = –1.556 (*p* = 0.122)
Courage	5.16 (1.255)	5.15 (1.227)	5.17 (1.285)	*t* = –0.076 (*p* = 0.939)
Perseverance	5.69 (1.409)	5.71 (1.273)	5.67 (1.511)	*t* = 0.155 (*p* = 0.877)
Honesty	5.56 (1.362)	5.37 (1.237)	5.70 (1.438)	*t* = –1.348 (*p* = 0.180)
Vitality	5.78 (1.139)	5.94 (1.056)	5.66 (1.190)	*t* = 1.372 (*p* = 0.173)
Love	6.09 (0.831)	**6.38 (0.844)**	5.87 (1.382)	*t* = 2.535 (*p* = 0.013)
Amability	6.41 (0.831)	6.50 (0.728)	6.34 (0.899)	*t* = 1.034 (*p* = 0.303)
Intelligence	5.84 (1.160)	5.85 (1.092)	5.83 (1.215)	*t* = 0.082 (*p* = 0.934)
Teamwork	6.09 (1.012)	**6.37 (0.768)**	5.89 (1.123)	*t* = 2.652 (*p* = 0.009)
Justice	5.92 (0.941)	5.88 (1.003)	5.94 (0.899)	*t* = –0.337 (*p* = 0.737)
Leadership	5.30 (1.346)	5.25 (1.203)	5.33 (1.452)	*t* = –0.318 (*p* = 0.751)
Forgiveness	5.43 (1.226)	5.40 (1.376)	5.46 (1.112)	*t* = –0.236 (*p* = 0.813)
Humility	5.99 (0.940)	6.06 (0.850)	5.94 (1.006)	*t* = 0.666 (*p* = 0.507)
Prudence	5.68 (1.235)	5.69 (1.147)	5.67 (1.305)	*t* = 0.092 (*p* = 0.927)
Selfcontrol	5.30 (1.390)	5.48 (1.244)	5.17 (1.484)	*t* = 1.218 (*p* = 0.226)
Wonder	5.85 (1.034)	5.83 (1.098)	5.87 (0.992)	*t* = –0.234 (*p* = 0.815)
Gratitude	6.58 (0.641)	6.44 (0.777)	6.69 (0.498)	*t* = –1.977 (*p* = 0.051)
Optimism	5.31 (1.361)	5.44 (1.259)	5.21 (1.433)	*t* = 0.914 (*p* = 0.362)
Sense of humor	6.08 (0.992)	6.15 (0.872)	6.03 (1.076)	*t* = 0.688 (*p* = 0.493)
Spirituality	4.31 (1.855)	4.44 (1,765)	4.21 (1.925)	*t* = 0.670 (*p* = 0.504)
MCSP	7.47 (1.187)	7.42 (1.242)	7.50 (1.152)	*t* = –0.353 (*p* = 0.725)
PMCS	10.66 (1.519)	10.25 (1.846)	**10.97 (1.142)**	*t* = –2.487 (*p* = 0.015)

*CTR, corrected typified residuals; those less than –1.96 or greater than 1.96 were considered significant. The groups from among the categorical variables in which the CTRs were significant are shown in bold. When the post hoc tests in the quantitative variables were significant, we have indicated the highest scoring group; n, sample; M, average; SD, standard deviation; χ^2^, Pearson chi-squared test; t, Student’s t-test; GACS-24, Global Assessment of Character Strengths-24; PIL, purpose in life.*

### Psychopathological Variables

In terms of psychopathology (Table 3), compared to nursing students, medical students had lower mean BAI scores (*M* = 8.24; *SD* = 7.767 vs. *M* = 11.19; *SD* = 8.381; t = 2.005; *p* = 0.047) and less presented an anxiety disorder (42.9%; *n* = 30 vs. 63.5%; *n* = 33; χ2 = 5.072; *p* = 0.024). Medical students (*M* = 5.33; *SD* = 4.596) also had lower ASD scores than nursing students (*M* = 7.29; *SD* = 4.820; t = 2.281; *p* = 0.024) and a lower proportion of ASD (17.1%; *n* = 12 vs. 36.5%; *n* = 19; χ2 = 5.922; *p* = 0.015). Finally, medical students (*M* = 24.46; *SD* = 16.582) had less psychopathological symptoms than nursing students (*M* = 31.85; *SD* = 18.375; t = 2.324; *p* = 0.022).

**TABLE 3:** Psychopathology in students.

	Total *N* = 122	Nursing students *n* = 52	Medical students *n* = 70	
	% (*n*) / *M* (*SD*)	% (*n*) / *M* (*SD*)	% (*n*) / *M* (*SD*)	*t* / χ^2^(*p*) *post hoc*/CTR
BAI	9.50 (8.133)	**11.19 (8.381)**	8.24 (7.767)	*t* = 2.005 (*p* = 0.047)
Anxiety yes	51.6 (63)	**63.5 (33)**	42.9 (30)	χ^2^ = 5.072 (*p* = 0.024) 2.3/–2.3
BDI-II	7.96 (6.329)	9.17 (6.116)	7.06 (6.377)	*t* = 1.844 (*p* = 0.068)
Depression yes	15.6 (19)	21.1 (11)	11.4 (8)	χ^2^ = 2.146 (*p* = 0.143)
AS score	6.16 (4.773)	**7.29 (4.820)**	5.33 (4.596)	*t* = 2.281 (*p* = 0.024)
Acute stress yes	25.4 (31)	**36.5 (19)**	17.1 (12)	χ^2^ = 5.922 (*p* = 0.015) 2.4/–2.4
DAST-10	0.20 (0.616)	0.29 (0.776)	0.14 (0.460)	*t* = 1.206 (*p* = 0.232)
Drugs yes	13.1 (16)	15.4 (8)	11.4 (8)	χ^2^ = 0.410 (*p* = 0.522)
AUDIT	3.32 (2.785)	3.38 (3.504)	3.27 (2.126)	*t* = 0.206 (*p* = 0.837)
Alcohol yes	14.8 (18)	17.3 (9)	12.9 (9)	χ^2^ = 0.470 (*p* = 0.493)
Psychopathology	27.61 (17.680)	**31.85 (18.375)**	24.46 (16.582)	*t* = 2.324 (*p* = 0.022)
Mental disorder yes	66.4 (81)	69.2 (36)	64.3 (45)	χ^2^ = 0.327 (*p* = 0.567)

*CTR, corrected typified residuals; those less than –1.96 or greater than 1.96 were considered significant. The groups from among the categorical variables in which the CTRs were significant are shown in bold. When the post hoc tests in the quantitative variables were significant, we have indicated the highest scoring group; n, sample; M, average; SD, standard deviation; χ^2^, Pearson chi-squared test; t, Student’s t-test; BAI, Beck Anxiety Inventory; BDI-II, Beck Depression Inventory-II; AS, Acute Stress; DAST-10, Drug Abuse Screening Test; AUDIT, Alcohol Use Disorders Identification Test.*

### Linear Regressions and Data Modeling

Table 4 shows the results of the linear regressions, which allowed us to predict psychopathological variables based on the SARS-CoV-2 exposure as well as PIL, character strengths and moral courage variables. The BAI score was predicted by exposure of family and friends to the virus, GACS-24 spirituality, PIL, and GACS-24 honesty. The BDI-II score was predicted by PIL, while that of the ASD could be predicted by occupation. The AUDIT score was predicted by being a smoker, age, and the sum of character strengths from the GACS-24. Finally, total psychopathology was predicted with PIL, occupation, and the exposure of family and friends.

**TABLE 4:** Significant odds ratios of linear regression models predicting the presence of psychopathology.

Response	Predictors	OR (95% CI)	*P*-value
**BAI**	Exposure of family/friends	4.013 (1.806, 6.220)	<0.001
	GACS-24 Spirituality	1.047 (0.311, 1.783)	0.006
	PIL	–0.180 (–0.276, –0.084)	<0.001
	GACS-24 Honesty	–1.144 (–2.140, –0.149)	0.025

**BDI-II**	PIL	–0.201 (–0.272, –0.130)	<0.001

**ASD**	Occupation (nursing student)	–1.960 (–3.661, –0.259)	0.024

**AUDIT**	Smoker	1.749 (0.507, 2.991)	0.006
	Sum of strengths GACS-24	–0.035 (–0.065, –0.004)	0.027
	Age	–0.133 (–0.231, –0.034)	0.009

**Total PSY**	Exposure of family/friends	5.189 (0.546, 9.382)	0.029
	PIL	–0.394 (–0.604, –0.184)	<0.001
	Occupation (nursing student)	–8.304 (–14.254, –2.354)	0.007

predictor variables plus age, sex, spirituality, psychological/psychiatric treatment during the pandemic, physical illness, and psychiatric history were all entered into all the forward models. BAI: Beck Anxiety Inventory; BDI-II: Beck Depression Inventory-II; ASD: Acute Stress Disorder; AUDIT: Alcohol Use Disorders Identification Test; PSY: psychopathology.

**FIGURE 1:**
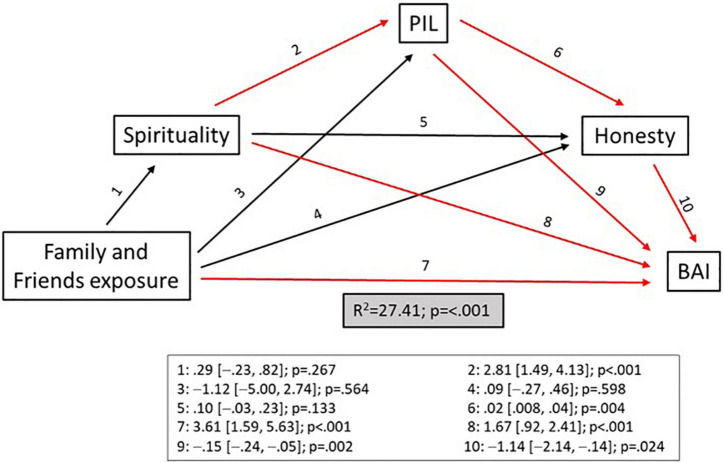
Explanatory model between purpose in life, spirituality, honesty, exposure, of family and friends, and the Beck’s Anxiety Inventory. Significant relationships are marked in red. BAI, *Beck Anxiety Inventory*; PIL, purpose in life.

Finally, Figure 1 shows a model describing the predictor effect of family and friends exposure to SARS-CoV-2, PIL, spirituality, and honesty in BAI. Family and friends exposure to SARS-CoV-2 (B[95% CI] = 3.61 [1.59, 5.63]; *p* < 0.001), PIL (B[95% CI] = –0.15 [–0.24, –0.05]; *p* = 0.002), spirituality (B[95% CI] = 1.67 [0.92, 2.41]; *p* < 0.001), and honesty (B[95% CI] = –1.14 [–2.14, –0.14]; *p* = 0.024) all directly affected BAI. In addition to a direct effect on anxiety, PIL also had an indirect effect through honesty (B[95% CI] = 0.02 [0.008, 0.04]; *p* = 0.004), while spirituality had an indirect effect through PIL itself (B[95% CI] = 2.81 [1.49, 4.13]; *p* < 0.001).

## Discussion

This is the first work to evaluate the effect of the COVID-19 pandemic on the development of psychopathology in health sciences students while also considering the effects exerted by PIL, character strengths and moral courage.

In our work the more exposure of family and friends of the health science students, the greater their risk of developing anxiety. Recent studies have shown that exposing family to COVID-19 was the main reason why healthcare workers did not go to work (33), being also the most important factor for the development of anxiety, depression and acute stress (11).

Previous studies have already shown the ability of PIL to positively moderate the prevention of psychiatric disorders (34, 35). A high PIL score reduces the likelihood of the appearance of anxiety (36, 37). In fact, PIL can affect anxiety both directly and indirectly, through honesty (Figure 1). In addition, and in line with previous research (38), a high PIL is also protective against the onset of depression. Taking all of this into account in medical and nursing students, it is worth highlighting the protective role that a high PIL has in terms of the appearance of anxiety, depression and total psychopathology.

On the contrary, intense spirituality is associated with lower subjective wellbeing in medical students (39) and can increase the levels of anxiety and depression (40–42), also among nursing personnel (43). However, other studies have opposite conclusions, stating that spirituality protects against the appearance of psychopathology (44–46). One of the possibilities for which this phenomenon could occur is “spiritual distress,” a state of suffering related to an impaired ability to experience purpose/meaning in life through connection with oneself, others, the world or a higher being (47). Spiritual distress frequently occurs in response to changes in health and life processes that disrupt a person’s sense of purpose/meaning, just as it might happen in a pandemic. In fact, there are studies that linked participation in stressful events with increased spiritual distress and psychopathology (48). Thus, intense spirituality would act in two different ways: (1) Boosting the PIL and enhancing its protective role; (2) Acting as a vulnerability factor through the concept of spiritual distress (Figure 1).

Opposite to prior studies (11), no significant results have been found regarding the predictor role of moral courage. This could be explained because the health sciences students have not yet worked and therefore did not experience moral distress from it (difference between an individual’s moral expectations and the behavior they are actually able to implement) which is the responsible of developing psychopathology (49).

To sum, our work indicated that anxiety levels can predicted by the degree of family and friends’ exposure to SARS-CoV-2, PIL, honesty and the students’ level of spirituality (Figure 1).

Our research showed that nursing students had a higher incidence of anxiety, acute stress and total psychopathology, as seen prior to the pandemic (4). In fact, occupation (nursing) have been linked to higher risk of acute stress levels, coinciding these findings with the results of recent works (50) that demonstrated a high prevalence of acute stress among nursing personnel. Nevertheless, being a medical student was a protective factor against total psychopathology. Although no previous research has compared the profile of both these groups of students, we believe that our results should be considered based on the differences in the academic plan and the vocational peculiarities of the medical and nursing professions. Medicine is characterized by an analytical approach, oriented toward diagnosis and treatment, with more distant treatment, while nursing is characterized by close contact with the patient, taking an emotional approach that requires empathy. This may be associated with the greater involvement of nursing staff in the processes of the patients they treat, meaning that they integrate part of the suffering of others and thereby themselves tend to develop symptoms of anxiety, stress, or depression.

This theory would be reinforced by the finding that occupation (nursing) can predict high levels of acute stress and the better results for GACS-24 teamwork and love item obtained by nursing students. As an alternative hypothesis, medical students may have greater social desirability than nursing students meaning that they therefore tend to minimize psychopathology. Indeed, previous studies have shown an inverse relationship between social desirability and psychopathology (51). However, other elements could also influence the development of mental pathologies in students. For example, discontinuity in the educational process, conversion toward solely digital teaching models, and the lack of resources provided by academic institutions are factors that affect students and may have increased their levels of anxiety and stress (52). In addition, the suspension of clinical practices during the pandemic, and at a time of increased healthcare pressure in hospitals, in parallel to the maintenance of students’ academic obligations during the periods of confinement, could also have contributed to the development of symptoms (53).

Therefore, it is advisable to pay attention to the education of health sciences students as it could affect their future mental health. Likewise, not addressing the psychopathology they present as students would have a negative impact on their future job performance (54). One way to prevent this psychopathology would be to incorporate to their student training workshops and seminars aimed at reinforcing their purpose in life through individual introspection exercises on personal goals and passions (55).

Finally, we must mention the main limitations of this study. Firstly, this work was carried out at a single Health Sciences Faculty, which may reduce its external validity compared to multicenter studies. Secondly, when weighing up the impact of the COVID-19 pandemic we must consider the cumulative incidence in each region or country, which was lower in the province where the study was carried out compared to other areas in Spain and other countries. Sociodemographic differences between both groups of students should also be considered because these could influence the presence of psychopathology. Thirdly, because of the cross-sectional nature of this study, we cannot state with certainty that the psychopathological results we found responded exclusively to the COVID-19 pandemic and the predictor variables. The intentional sampling used in the recruitment should also be considered as a possible methodological weakness.

## Conclusion

This study shows that the COVID-19 pandemic impacted the development of psychopathology in health sciences students. Specifically, exposure of the students’ family and friends to SARS-CoV-2 favored the appearance of symptoms of anxiety. Studying medicine was a protective factor against total psychopathology while being a nursing student was associated with high levels of acute stress. In contrast, honesty had a preventing role in the onset of anxiety and a high PIL was protective against the appearance of anxiety, depression, and total psychopathology.

## Data Availability Statement

The raw data supporting the conclusions of this article will be made available by the authors, without undue reservation.

## Ethics Statement

The studies involving human participants were reviewed and approved by the Investigation Commission at the Provincial Hospital Consortium in Castellon (ref. A-15/04/20) and the Clinical Research Ethics Committee of the Cardenal Herrera-CEU University (ref. CEI20/068). The patients/participants provided their written informed consent to participate in this study.

## Author Contributions

IE, AB, and GH: conceptualization. IE and IA: data curation. AB: formal analysis. MP, DP, and VQ: investigation. IE, MP, DP, and VQ: draft preparation. IE, MP, DP, VQ, AB, IA, and GH: review and editing. GH: funding acquisition. All authors have read and agreed to the published version of the manuscript.

## Conflict of Interest

The authors declare that the research was conducted in the absence of any commercial or financial relationships that could be construed as a potential conflict of interest.

## Publisher’s Note

All claims expressed in this article are solely those of the authors and do not necessarily represent those of their affiliated organizations, or those of the publisher, the editors and the reviewers. Any product that may be evaluated in this article, or claim that may be made by its manufacturer, is not guaranteed or endorsed by the publisher.
